# Supplementary material for: COVID-19 Vaccine and Death: Causality Algorithm According to the WHO Eligibility Diagnosis
Source: Diagnostics (Basel). 2021 May 26;11(6):955. doi: 10.3390/diagnostics11060955 (PMC8229116; doi:10.3390/diagnostics11060955)
Supplement: Supplementary file 1 [file diagnostics-11-00955-s001.zip › diagnostics-1216623-supplementary.pdf]

APPENDIX 1. Anti SARS-CoV-2 VACCINES

We have analyzed the interim recommendations of WHO for the use of the Pfizer–BioNTech COVID-19 vaccine[1], Moderna mRNA-1273 vaccine against COVID-19[2], the AZD1222 (ChAdOx1-S (recombinant)) vaccine against COVID-19 developed by Oxford University and AstraZeneca[3], and the Janssen Ad26.COV2.S (J&J)[4].

The Pfizer–BioNTech COVID-19 vaccine, BNT162b2, is an mRNA vaccine encoding a P2 mutant spike protein (PS 2) and formulated as an RNA–lipid nanoparticle of nucleoside-modified mRNA (modRNA). Its use is general recommended for subjects aged 16 years and above. The recommended schedule is two doses with an interval of 21–28 days. The most common solicited adverse reactions in participants 16 years of age and older included pain at the injection site, fatigue, headache, muscle pain, etc… The severe adverse effects are lymphadenopathy, bell's palsy, and allergic reactions [1,5].

The Moderna COVID-19 vaccine contains a synthetic mRNA (single-stranded, 5'-capped) encoding the prefusion-stabilized spike glycoprotein (S) of SARS-CoV-2 virus. The vaccine also contains the following ingredients: lipids (SM-102, 1,2-dimyristoyl-rac-glycero-3-methoxypolyethylene glycol-2000 (PEG2000-DMG), cholesterol, and 1,2-distearoyl-sn-glycero-3-phosphocholine (DSPC)), tromethamine, tromethamine hydrochloride, acetic acid, sodium acetate, and sucrose. It is administered intramuscularly as a series of two doses (0.5 ml each), given 28 days apart. During the clinical trials, the symptoms described related to the vaccine administration were pain at the injection site, fatigue, headache, myalgia, etc… Moreover, although rarely, adverse events of special interest were lymphadenopathy-related events, Bell's palsy, and hypersensitivity-related events[2,6].

AZD1222 vaccine is a monovalent vaccine composed of a single recombinant, replication-deficient chimpanzee adenovirus vector encoding the S glycoprotein of SARS-CoV-2 (ChAdOx1-S (recombinant). The recommended schedule is two doses administered with an interval of 4-12 weeks. Its use is general recommended for subjects aged 18 years and above, even if considering severe adverse reaction (the so-called thrombotic thrombocytopenia post-vaccination) temporarily related with the vaccine administration, each country has chosen independently [3,7].

Janssen Ad26.COV2.S (COVID-19) vaccine is a recombinant, replication-incompetent adenovirus serotype 26 (Ad26) vector encoding a full-length and stabilized SARS-CoV-2 spike protein. This vaccine does not contain adjuvants, preservatives, materials of animal origin, or fetal tissue. It is suggested for persons aged 18 years and above, and it may be administrated in a single dose. Apart from the classic symptoms after vaccination (fever, headache, diarrhea, vomiting), in the clinical trials no severe adverse effects were reported, even if recently, several cases of thrombosis are described[4,8].

References

1. WHO Interim recommendations for use of the Pfizer–BioNTech COVID-19 vaccine, BNT162b2, under Emergency Use Listing Available online: https://www.who.int/publications/i/item/WHO-2019-nCoV-vaccines-SAGE_recommendation-BNT162b2-2021.1 (accessed on Apr 14, 2021).
2. WHO Interim recommendations for use of the Moderna mRNA-1273 vaccine against COVID-19 Available online: https://www.who.int/publications/i/item/interim-recommendations-for-use-of-the-moderna-mrna-1273-vaccine-against-covid-19 (accessed on Apr 14, 2021).
3. WHO Interim recommendations for use of the AZD1222 (ChAdOx1-S (recombinant)) vaccine against COVID-19 developed by Oxford University and AstraZeneca Available online: https://www.who.int/publications/i/item/WHO-2019-nCoV-vaccines-SAGE_recommendation-AZD1222-2021.1 (accessed on Apr 14, 2021).
4. WHO Interim recommendations for the use of the Janssen Ad26.COV2.S (COVID-19) vaccine: interim guidance Available online: https://apps.who.int/iris/handle/10665/340203 (accessed on Apr 14, 2021).
5. WHO Background document on mRNA vaccine BNT162b2 (Pfizer-BioNTech) against COVID-19 Available online: https://www.who.int/publications/i/item/background-document-on-mrna-vaccine-bnt162b2-(pfizer-biontech)-against-covid-19 (accessed on Apr 14, 2021).
6. WHO Background document on the mRNA-1273 vaccine (Moderna) against COVID-19 Available online: Background document on the mRNA-1273 vaccine (Moderna) against COVID-19 (accessed on Apr 14, 2021).
7. WHO Background document on the AZD1222 vaccine against COVID-19 developed by Oxford University and AstraZeneca Available online: Background document on the AZD1222 vaccine against COVID-19 developed by Oxford University and AstraZeneca (accessed on Apr 14, 2021).
8. WHO Background document on the Janssen Ad26.COV2.S (COVID-19) vaccine: Background document to the WHO Interim recommendations for use of Ad26.COV2.S (COVID-19) vaccine Available online: https://www.who.int/publications/i/item/WHO-2019-nCoV-vaccines-SAGE-recommendation-Ad26.COV2.S-background-2021.1 (accessed on Apr 14, 2021).
